# Incidental Breast Findings on Chest CT: Rates of Report and Follow‐Up

**DOI:** 10.1155/rrp/8477697

**Published:** 2026-06-25

**Authors:** Jessica Hui, Fabiana Chacur Policeni, Rodrigo Moreira Bello, Su Kim Hsieh

**Affiliations:** ^1^ Department of Radiology, University of Iowa Hospitals and Clinics, 200 Hawkins Dr, Iowa City, 52242, Iowa, USA, uihealthcare.org

**Keywords:** breast neoplasm, chest CT, incidental findings

## Abstract

**Objective:**

This study aims to determine the incidence and types of breast findings detected on chest CTs at our institution and assess rates of subsequent follow‐up imaging and breast cancer screening.

**Methods:**

A retrospective review of all chest CTs performed at our institution over a 2‐year period (1/1/2022–1/1/2024) was conducted, demonstrating 1540 patients above the age of 40 with incidental breast findings identified using a text search of the reports, and follow‐up recommendations were documented. Clinical outcomes were analyzed for cases with recommended dedicated breast imaging.

**Results:**

Incidental breast findings were detected in 14.3% of chest CTs in women above or at the age of 40. Further breast imaging was recommended in 54 cases, with 21 cases having associated dedicated breast imaging performed. Of these, corresponding dedicated workup revealed predominantly benign findings, with 82% representing benign or likely benign etiologies and only 4 cases representing malignancy. The likelihood of performing follow‐up imaging was influenced by the language used in the report, with a 40% completion rate for studies using definitive wording as compared to 0% for optional wording.

**Conclusion:**

There is a considerable rate of incidental breast findings visualized on chest CTs, which are predominantly benign. However, as there is a potential for visualization of malignant lesions, standardized reporting and follow‐up protocols are essential to ensure proper evaluation and management. Further research is needed for evaluating and standardizing the potential use of chest CTs for assessing incidental breast lesions.

**Key Findings**
• Incidental breast findings were identified in 14.3% of chest CTs performed in women of the age for screening mammography. Of these, 5.4% required further dedicated breast imaging, revealing predominantly (82%) benign findings.
• There are variable rates of follow‐up dedicated breast imaging performed depending on report verbiage, which emphasizes the need for standardized follow‐up protocols as the expected rate of inclusion of breast tissue on CT increases with increased utilization of chest CT across all indications, including LDCT lung cancer screening.

## 1. Introduction

Breast cancer is a leading cause of morbidity and mortality in women and is the most common cancer in women worldwide, with almost 13% of women being diagnosed with breast cancer during their lifetime [1]. Currently, mammography/contrast‐enhanced mammography (CEM), ultrasonography, and magnetic resonance imaging provide the bulk of studies ordered for screening or diagnostic evaluation of breast findings, following current ACR guidelines for annual breast screening to consist of mammography with or without digital breast tomosynthesis, CEM, and/or MRI [2, 3]. While not currently the initial consideration for evaluation of or screening for breast cancers, as chest CT sensitivity and specificity are significantly lower as compared to MRI for evaluation of breast lesions, chest CTs are regularly performed for evaluation of a wide spectrum of findings, ranging from acute pathologies to other diseases or malignancies, including screening for lung cancers with partial or complete incidental inclusion of the bilateral breast tissue within the field of view during a standard protocol. At this time, it is recommended that diagnostic mammography and/or targeted ultrasound be performed for evaluation of incidental breast abnormalities seen on other modalities [4]. As such, chest CTs have been shown to provide a potential form of evaluation of breast tissue without causing additional radiation dose [2]. Additionally, chest CT has a significant role in evaluating large and locoregional invasive breast tumors, such as chest wall invasion, mediastinal extension, bone involvement, and pulmonary metastatic disease [5, 6]. Although still in the experimental phase, prior research also demonstrates the utility of dedicated contrast‐enhanced breast CT for identifying malignant breast masses [6–8].

Currently, there is a wide range of reported incidence of breast findings on chest CT in the literature, ranging from 0.3% to 8%, though this is still believed to be underreported [8, 9]. Thus far, though there have been several studies which have begun to explore the use of chest CT to assess breast cancer risk factors such as breast density [5], there has been a relative dearth of literature regarding the incidence and type of breast findings noted on chest CTs, and the potential for evaluation of breast tissue on CT has not yet been completely explored.

Furthermore, there is a lack of standardization of verbiage between thoracic radiologists and breast radiologists, with Fleischner criteria describing masses as “any circumscribed lesion greater than 30 mm in diameter” [10], whereas BI‐RADS criteria describe a mass as a “three‐dimensional, space‐occupying lesion with completely or partially convex borders,” with BI‐RADS also typically providing a structured set of recommendations for next steps [2, 11]. Additionally, breast findings on CT are often described as nodules instead of masses, although this term does not exist in the BI‐RADS lexicon. As such, these discrepancies between descriptors and verbiage for recommendations may lead to difficulty on the part of the ordering provider when it comes to appropriate follow‐up. Therefore, in this study, we aim to better characterize and describe not only the incidence of breast findings on chest CT at our institution but also the categories of findings, range of significance, and rates of follow‐up performed, in order to better understand both the types of findings, verbiage used to describe them, and subsequent significance on follow‐up. Wording of the report impression was also noted to improve understanding of reporting language and apparent strength of recommendation would affect follow‐up completion rate. Ultimately, our goal is to improve awareness of reporting breast findings on nondedicated imaging, and to show that within a single institution, there may be a wide variety of descriptors provided which do not follow a structured lexicon.

## 2. Methods

### 2.1. Ethics Statement

This retrospective review study at a single academic medical center with associated outpatient tertiary cancer center was approved by our Institutional Review Board (IRB#200903778), and the requirement for written consent from the patients was waived. Patient medical records were reviewed in compliance with Health Care Portability and Accountability Act guidelines.

### 2.2. Data

**FIGURE 1:**
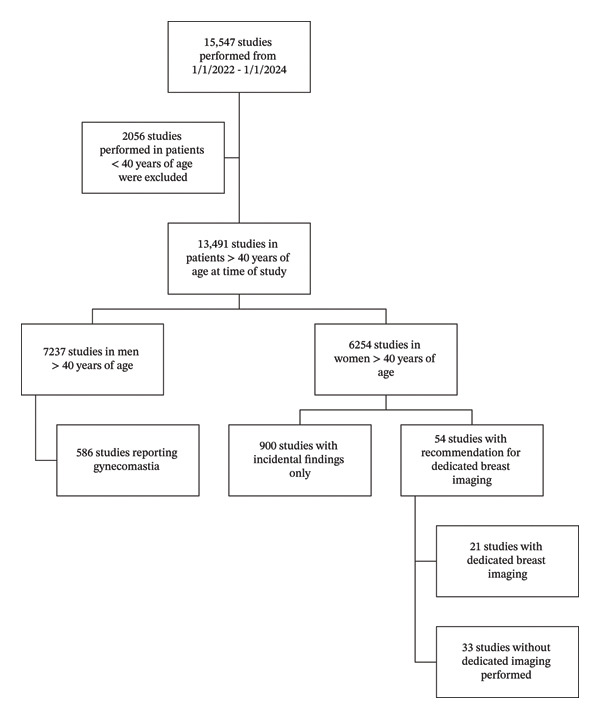
Schematic flowchart of study selection, including initial search criteria and subsequent study inclusion.

**FIGURE 2:**
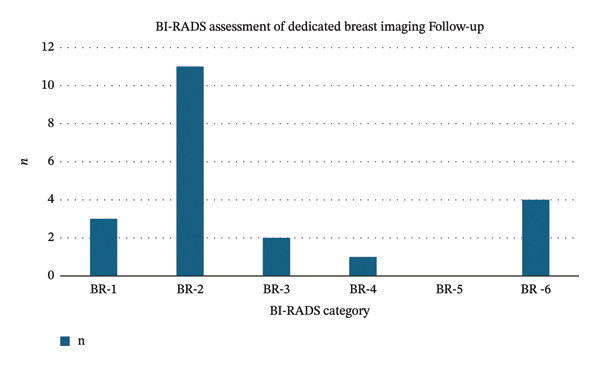
BI‐RADS categorization of findings on corresponding dedicated breast imaging.

A retrospective review of all chest CTs (with contrast, without contrast, low‐dose lung cancer screenings, and CT esophageal leak studies) performed within a 2‐year period from 1/1/2022 through 1/1/2024 at our quaternary care facility conducted and yielded 15,547 studies, which included an age range of 0.1–103 years of age. These were performed across six different scanners on Siemens Somatom FORCE, NAEOTOM, and Somatom Definition AS 64 devices. Images were then interpreted and reported by Thoracic Imaging attending radiologists at the University of Iowa. Those with age less than 40 were excluded, yielding a total of 13,491 studies (Figure 1). The body of the report text was then searched for incidental breast findings by searching for the phrases “breast,” “gynecomastia,” and “dedicated breast imaging” or “diagnostic breast imaging.” Given the high rate of gynecomastia, data for men and women were reported separately. All reports containing mention of “breast” were reviewed for descriptions of the breast findings in question, with verbiage as described in Tables 1 and 2, as well as for recommendations contained within the impression text. The report and impressions for studies recommending further follow‐up were further individually reviewed for imaging descriptors and recommendation phrasing, with absolute verbiage being noted as direct if any form of “recommend correlation with dedicated breast imaging” was used. Qualifying or optional verbiage such as “if clinically indicated,” “unless already performed,” or “correlate with prior [dedicated breast] imaging if available” was also noted. Age, gender, indication for study, and history of cancer(s) were also noted for this subset of studies. There was a wide variety of indications for these studies, ranging from the acute setting for diagnosis of infection or infection follow‐up, post‐traumatic, or postsurgical workup, to surveillance of lung nodules or known history of lung malignancies. Descriptive statistical analysis was performed for summarization of data and graphical representation of distribution of findings across general CT descriptors and BI‐RADS categories (Tables 1 and 2, Figure 2). Additional statistical analysis was limited secondary to low *n*. Subsequent dedicated breast follow‐up imaging was performed with either screening or diagnostic mammography, with supplemental ultrasonography or MRI if indicated. Malignant outcomes for findings listed as BI‐RADS 6 were pathology‐proven on prior biopsy. Imaging surveillance was performed for BI‐RADS 3 findings, and one BI‐RADS 4 finding was biopsied and pathology‐proven fibroadenoma.

**TABLE 1:** General category of CT breast findings as reported in the impression text based on best judgment of impression text.

Types of CT findings	*n*	*n* (%)
Mass/nodule	28	51.8
Asymmetric tissue/thickening	15	27.8
Postsurgical changes	7	12.9
Calcifications	3	5.6
Nipple retraction	1	1.8

*Note:* The total number of these findings is noted as well as the percentages.

**TABLE 2:** Chest CT and correlates as identified on corresponding dedicated breast imaging studies for each individual patient.

CT findings	Correlated diagnosis
Rim enhancing area	BI‐RADS 2, fat necrosis
Breast nodule	BI‐RADS 2, hemorrhagic cyst
Breast lesion	BI‐RADS 2, known seroma
Postoperative appearance	BI‐RADS 2, postsurgical changes
Discrete round cystic mass	BI‐RADS 2, stable complicated cyst
Calcifications, asymmetric tissue	BI‐RADS 3, oval mass
Oval nodular lesions	BI‐RADS 3, oval masses
Indeterminate nodule	BI‐RADS 4, fibroadenoma (biopsy‐proven)
Spiculated lesion (left), tiny nodule (right)	BI‐RADS 6, bilateral invasive ductal carcinoma
Asymmetric tissue	BI‐RADS 6, known invasive ductal carcinoma
Soft tissue nodules	BI‐RADS 6, known invasive ductal carcinoma
Known breast mass	BI‐RADS 6, known invasive lobular carcinoma

*Note:* Findings on chest CT which did not demonstrate a correlate on corresponding dedicated breast imaging are not shown. Chest CT findings with verbiage as noted in the report text and the corresponding correlate as noted on subsequent dedicated breast imaging, with each line representing an individual patient.

## 3. Results

Our initial pool was composed of 13,491 chest CT exams performed within a 2‐year period within our institution, with 6254 (46%) studies having been performed on women. In the 7237 studies performed in men, 586 studies (8.1%) were noted to report gynecomastia without recommendation for further imaging evaluation. Of the studies performed in women, 900 studies noted benign findings only, resulting in an incidence of 14.3% for any breast finding noted on chest CT. These covered a wide range of categories including post‐traumatic or postsurgical findings such as breast implants which were not felt to require further evaluation by the interpreting radiologist. For these studies, no recommendation for further workup was given. Only 54 studies (5.9%) recommended additional correlation with dedicated breast imaging for further evaluation.

**FIGURE 3:**
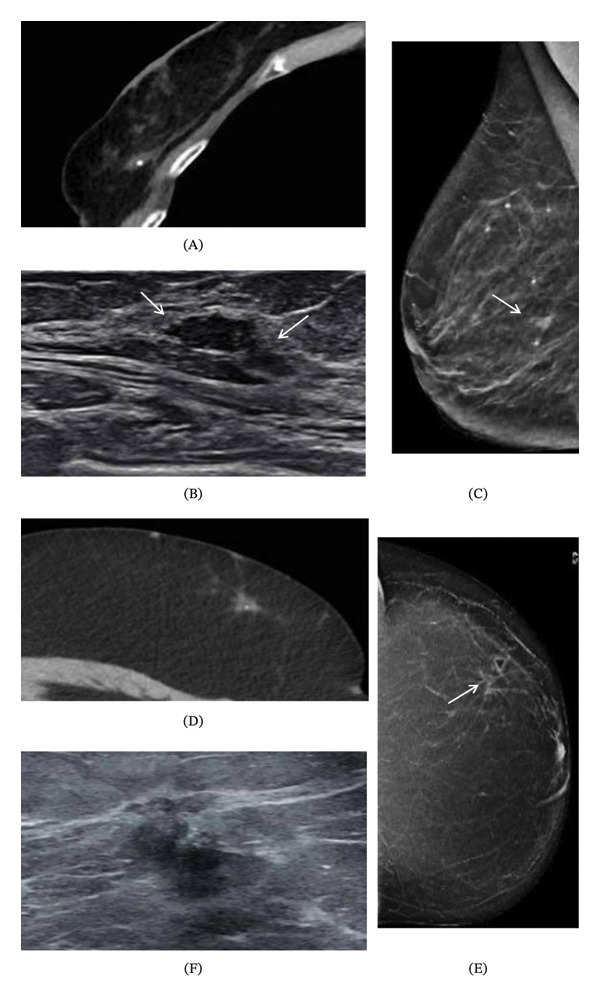
Known malignancies incidentally visualized on chest CT. (A) Chest CT demonstrating small soft tissue density oval mass (white arrow) in the right lower outer quadrant with biopsy clip in place, with biopsy results reported as consistent with invasive ductal carcinoma. (B) Prebiopsy ultrasound demonstrating a hypoechoic irregular mass without circumscribed margins which was felt to correspond with the CT finding. (C) Prebiopsy mediolateral oblique (MLO) view mammogram of the right breast demonstrating irregular mass at the 9 o’clock position (white arrow). (D) Chest CT demonstrating soft tissue density spiculated mass with associated punctate calcifications in the left breast upper outer quadrant (white arrow). (E) Grayscale ultrasound demonstrating horizontal irregular mass with spiculated margins and associated microcalcifications (white arrowhead) and internal vascularity on color Doppler (not shown). (F) MLO view mammogram of the left breast demonstrating irregular mass with spiculated margins and associated calcifications at the 1 o’clock position (white arrow) which corresponds to the patient palpable area at the site of the skin marker (curved arrow).

Of the 54 studies which recommended dedicated breast imaging for further evaluation or comparison with prior dedicated imaging if available, the mean age of patients at the time of study was 64.6 ± 13.8 years. Of these studies, 20 dedicated breast imaging studies which comprised a mix of screening and diagnostic mammography were performed at our hospital, and 1 had been performed externally. Of these 21 studies, 14 (67%) demonstrated no mammographic correlation of the CT findings or showed benign findings and were categorized as either BI‐RADS category 1 or 2 on follow‐up mammography. Of the remaining 7 studies with dedicated breast imaging, these ranged from BI‐RADS category 3 to 6 (Figure 2), with 4 of these studies representing known primary breast malignancy, and the majority of these cases having been previously biopsied as invasive ductal carcinoma (Figure 3). Ultimately, 17 of 21 studies (82%) were felt to represent benign or likely benign etiologies. There were several different CT findings described, with the most common CT breast finding being described as nodules/nodularity, masses, or asymmetric tissue/thickening representing over 75% of the findings (Table 1).

**FIGURE 4:**
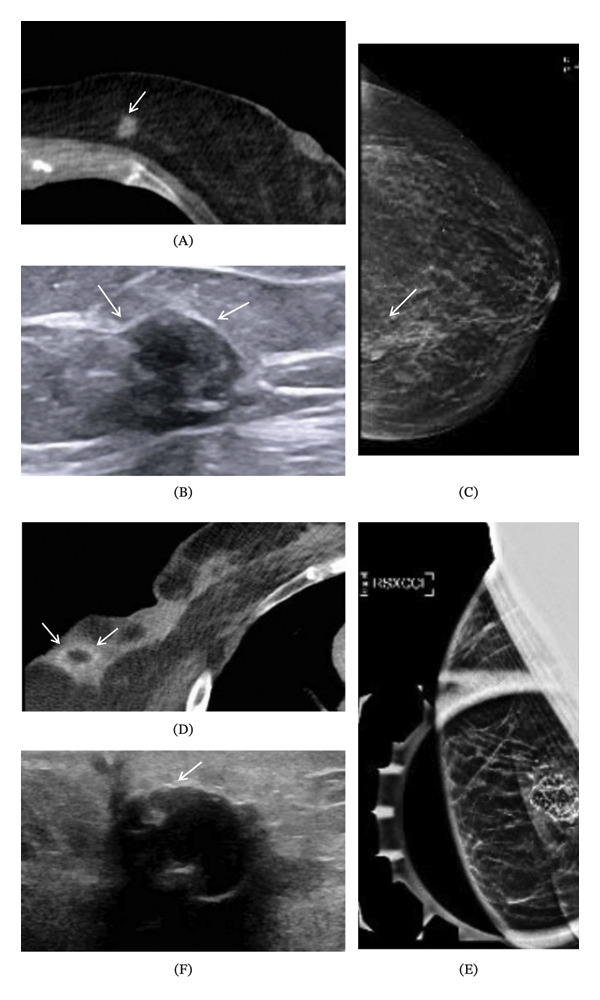
Benign findings incidentally noted on chest CT. (A) Chest CT on soft tissue window demonstrating soft tissue density oval mass in the posterior inner left breast (arrow). (B) Grayscale ultrasound demonstrating oval mass with heterogeneous echotexture and well‐circumscribed margins which was felt to likely correlate to the CT finding (arrows). This mass was subsequently biopsied and found to be consistent with fibroadenoma. (C) Mammogram of the left breast in the craniocaudal (CC) view demonstrates an oval mass with high density and circumscribed margins at the 9 o’clock position (arrow). (D) Chest CT on soft tissue window demonstrating a rim‐enhancing oval mass in the right lateral breast, closely associated with postsurgical changes of prior mastectomy. (E) Right laterally exaggerated craniocaudal (XCCL) spot compression view demonstrates a fat‐containing, round mass with circumscribed margins and associated dystrophic calcifications at the 9 o’clock position in the right lateral breast. (F) Grayscale ultrasound demonstrating mass closely associated with the mastectomy scar with echogenic rim (white arrow) and associated microcalcifications (arrowhead) and posterior shadowing, consistent with postsurgical fat necrosis.

The most common correlation between chest CT and dedicated breast imaging noted was an oval mass, which was found in 3 of the follow‐up mammographic studies and were categorized as BI‐RADS 2, 3, and 4, with the BI‐RADS 4 oval mass being biopsied and resulting as pathology‐proven fibroadenoma (Figures 3 and 4). The BI‐RADS 2 findings corresponded to hemorrhagic cysts, fat necrosis, and postsurgical changes including seroma or postsurgical scarring (Table 2), for which dedicated breast imaging was recommended for further characterization of presumed or clinically documented postsurgical changes. Of the two BI‐RADS 3 findings which corresponded to oval masses, stability was demonstrated at the initial ultrasound or MRI 6‐month follow‐up, and continued short‐term surveillance was recommended in keeping with BI‐RADS guidelines, although 2‐year stability has not yet been reached for these findings to allow for downgrade to BI‐RADS 2. The remaining findings noted on chest CT were not mentioned to have a corresponding finding on subsequent dedicated breast imaging. As noted in the reported impression text, there is a wide variety of verbiage used to describe the type of finding, with only two of the reports describing the shape of the mass or nodule (irregular) and only one report describing margins (spiculated).

We additionally noted that sorting by verbiage contained within the report impression text demonstrated that a direct recommendation of follow‐up dedicated breast imaging for further evaluation yielded a 53% completion rate (Table 3). Notably, there were also four studies for which follow‐up dedicated breast imaging was recommended, but prior imaging was subsequently obtained and deemed sufficient for comparison. Of note, contrast did not appear to yield an increased rate of dedicated follow‐up performed, with associated dedicated breast imaging having been completed in 40% (12 out of 30) of studies without contrast and only 29% (7 out of 24) of contrasted studies. The patient location at the time the study was ordered also did not lead to a significant difference in rate of follow‐up, with 34% (15 out of 44) of studies ordered in an outpatient setting, 20% (1 out of 5) of studies ordered in an inpatient setting, and 20% (1 out of 5) of studies ordered in an ED setting having corresponding dedicated breast imaging performed.

**TABLE 3:** Report recommendations and rates of subsequent dedicated imaging.

Report recommendations	Dedicated imaging performed	Dedicated imaging not performed
Follow‐up recommended	18	16
Follow‐up optional even without priors	0	16
Recommended for correlation, but optional if priors or externals available	0	4

*Note:* Absolute verbiage is noted as follow‐up recommended.

## 4. Discussion

Overall, our data indicate that incidental breast findings are found frequently in chest CTs, observed in 14.3% of women aged 40 and over, with 5.9% of these being actionable findings that warranted further dedicated breast imaging. While the majority of these actionable findings (82%) were ultimately determined to be benign or likely benign, the low rate of follow‐up for recommended dedicated breast imaging, coupled with the variable CT imaging descriptors and radiologist reporting verbiage, limits the definitive conclusions that can be drawn from these data regarding malignancy rates. Our study also noted an 8.1% incidence of gynecomastia in men that did not require further evaluation, which correlates with known chest CT and mammographic findings [12].

Both our study and published data demonstrate a wide range of incidental breast findings on chest CT, which run the gamut from benign to malignant. Benign findings have been shown to have some association with descriptors such as “round,” “lobulated,” or “indistinct” [8, 13, 14]. Additionally, calcifications visualized on chest CT have also been shown to be associated with benignity. As the spatial resolution of CT is lower in comparison to mammography, calcifications large enough to be detected on CT are typically macrocalcifications and thus demonstrate a high rate of being associated with benign findings [14, 15].

Of these incidental breast findings on chest CT, previous studies have shown that a range of 31%–70% of these lesions with features concerning enough to recommend dedicated breast follow‐up imaging have subsequently been shown to be malignant on further evaluation [5, 13]. Overall, irregular shape, noncircumscribed margins, and avid enhancement on CT have been shown to be suggestive of malignancy [5, 13, 14]. An additional recent study to evaluate the potential of CT features of incidental breast lesions for breast screening also noted that increased enhancement with a change of greater than 20 HU in combination with noncircumscribed margins provided a high diagnostic accuracy for malignancy of 97.7% [16]. Furthermore, lesions that have margins which may be described as spiculated on CT have also been shown to demonstrate a PPV of 76% for malignancy [14]. Adjunct findings such as invasion of the chest wall with involvement of the pectoralis muscle, overlying skin thickening, or enlarged axillary or internal mammary chain lymph nodes would also aid in supporting a diagnosis of an advanced primary malignancy [15, 16].

Overall, there has also been some overlap of classic findings for major subtypes of breast malignancy with the expected findings on dedicated breast imaging within the existing literature, suggesting that certain chest CT findings may predispose to a specific diagnosis. In parallel with mammographic and MRI findings, invasive ductal carcinoma has been shown to present as an irregular mass with spiculated margins, whereas invasive lobular carcinoma has been shown to present with asymmetric soft tissue densities with or without skin thickening. Mucinous neoplasms have likewise been shown in several cases to be described on CT as soft tissue masses with foci of lower attenuation [16–20].

Despite this inclusion of breast parenchyma in chest CTs and the potential significance of these findings, breast findings may frequently be overlooked or incorrectly evaluated in the report. Furthermore, even if breast findings are noted, no structured lexicon or recommendations exist for breast findings on CT, and consequently referring providers may not be as aware of next steps following reports of positive breast findings on chest CT. While our data suggest that when a recommendation for dedicated breast imaging was phrased with optional verbiage that a significantly lower percentage of follow‐up studies were performed, a recent study by Mannix et al. showed no difference in rates of follow‐up dedicated breast imaging depending on absolute versus optional verbiage [21, 22]. At our institution, studies where follow‐up is recommended and require action by the ordering provider are typically marked as attention needed, which will flag the study in the electronic medical record (EMR) system. These studies also frequently include direct communication with the ordering provider by the reading radiologist with confirmation of receipt by the ordering. As such, these factors potentially contribute to the difference in adherence to impression recommendations for dedicated follow‐up breast imaging.

Although no primary diagnosis of a new breast malignancy was found within our data despite the noted frequency of incidental breast findings on chest CT, this may be in part be due to the overall lower rate of follow‐up as well as the relatively low proportion of female patients within the age range for screening chest CTs. However, as the number of low‐dose screening chest CTs increases, the rate of incidentally noted breast findings may likewise be expected to increase if all recommended follow‐up is performed, which could suggest that a higher rate of incidentally detected breast neoplasms may be expected. As such, a standardized reporting lexicon for breast findings on chest CT has been suggested both to improve clarity of recommendations for further workup and to aid in understanding of the importance of completing follow‐up dedicated breast imaging.

One recent study explored this concept further to provide a prospective scoring system for these incidental breast findings, which they have titled as BARCS (breast assessment and recommendation CT score). This proposed scoring system was designed to correspond directly to the equivalent BI‐RADS assessment scores, with categories spanning from 0 to 2, as of that for screening mammography. The authors proposed that BARCS‐1 be used to describe chest CTs with negative breast findings and BARCS‐2 be used for benign findings such as simple cysts or well‐defined, encapsulated masses with coarse calcifications felt to be consistent with fibroadenoma, equivalent to that of BI‐RADS 1 and 2. Subsequent correlation with BI‐RADS scoring suggests a high rate of agreement between BARCS‐1 or 2 and BI‐RADS 1 or 2 findings [20, 23]. However, BARCS‐0 appeared to have a lower rate of agreement of 34%, as compared to the author‐reported concordant BI‐RADS scores of 0, 4, or 5. Although findings to be graded as BARCS score of 0 were not completely defined, examples given were findings which met the BI‐RADS descriptors for mass (3‐dimensional, space‐occupying lesion with convex borders) or as calcifications which were not clearly benign [24].

Currently, in the literature, there is a reported rate of follow‐up completion ranging from 8% to 37% for any radiology reports with a wide variety of contributing factors from both the patient and the ordering provider. These reported factors included patient loss to follow‐up, report verbiage, or failure to review radiology report recommendations versus incomplete understanding of significance of radiologic findings by the ordering provider [19, 20, 24]. A recent study in *AJR* reported on the potential efficacy of a human‐based system for tracking completion of follow‐up recommended by radiology report impressions, demonstrating improved rates of follow‐up when patient navigators reached out to ordering providers, with escalation to the patient’s primary care physician if no response was given by the initial ordering provider [25]. This proposal is also supported by another study in *AJR,* which reported a 30% increase in rates of follow‐up over all types of radiology reports when multistage human and EMR‐based tracking was implemented, with increasing escalation over a 3‐month period after unconditionally worded recommendations for timing of further follow‐up became overdue [26]. Additionally, there has been literature suggesting that the exam setting may influence follow‐up rates, with studies performed in inpatient and ED settings receiving less follow‐up than outpatient settings [27, 28].

Potential suggestions for optimizing further management and improving patient care and outcomes may include a combination of the current approaches available at major academic centers and those proposed within the literature. As supported by both our data and the existing literature on improving follow‐up, a first step could be to specify standardized unconditional or absolute wording within imaging report impressions for further workup, so as to reduce uncertainty for the ordering clinician regarding the radiologist’s recommendations [14, 15, 21]. This could potentially be accomplished by following a recently published taxonomy of guidelines for report impressions and increasing the rate of complete recommendations, which was defined by the authors to include modality, time frame, and reasoning for the finding in question. Ambiguous, conditional, or alternative wording for report impression recommendations were found to result in a significantly lower rate of completion [29–31]. Brief review of the literature also suggests that further improvement in compliance could also potentially be achieved with integrating an automated flagged reminder within the EMR or multistage human‐based EMR tracking as suggested by multiple studies in the current literature to decrease provider‐related rates of noncompliance, with responsibility to be assigned to a single healthcare provider as opposed to multiple separate providers [22–26, 31].

This study demonstrates several limitations, which primarily stem from our small sample size of studies with adequate dedicated breast imaging follow‐up, which lead to limited ability to analyze mammographic correlates and potential descriptors which would provide statistically significant associations with malignancy. Limitations also include retrospective study at a single institution, which limits the generalizability of the findings provided. Of note, there were relatively few incidental breast findings noted on low‐dose lung cancer screening chest CTs performed at our institution, which may be expected to decrease the rate of incidental findings noted, particularly in the female population. Data collection additionally did not account for whether the ordering provider noted follow‐up was not necessary or did not align with patient goals of care, which may have influenced the reported follow‐up rates.

As a result, although there are significant limitations to generalizability of our findings and application outside of this academic center, this study draws attention to the relatively high incidence of breast findings within chest CTs and to the low rate of dedicated breast imaging follow‐up performed following the initial CT report recommendation, which may be in part due to the variability in verbiage used. Increasing awareness of the potential significance of breast findings and differences in lexicon applied to describing incidental breast findings visualized on chest CT for both ordering providers and radiologists may improve ordering provider understanding of the report impression, especially with a view to improving recommendations and appropriate follow‐up, whether imaging or clinical. Next steps could include expanding assessment of absolute versus optional verbiage when providing strength of recommendation, as well as aiming to standardize reporting of breast findings on CT with a more structured lexicon in order to allow for improved understanding of further imaging to obtain for ordering providers, with a plan to repeat assessment to assess impact on follow‐up.

## Funding

No funding was received for this manuscript.

## Conflicts of Interest

The authors declare no conflicts of interest.

## Data Availability

The data that support the findings of this study are available from the corresponding author upon reasonable request.
